# Predicting Low-Cycle Fatigue Life Using New Energy-Based Fatigue Damage Measures

**DOI:** 10.3390/ma19020352

**Published:** 2026-01-15

**Authors:** Stanisław Mroziński, Michał Piotrowski, Władysław Egner, Halina Egner

**Affiliations:** 1 Faculty of Mechanical Engineering, Bydgoszcz University of Science and Technology, Al. Prof. S. Kaliskiego 7, 85-796 Bydgoszcz, Poland; stanislaw.mrozinski@pbs.edu.pl (S.M.); michal.piotrowski@pbs.edu.pl (M.P.); 2 Faculty of Mechanical Engineering, Cracow University of Technology, Al. Jana Pawła II 37, 31-864 Krakow, Poland; wladyslaw.egner@pk.edu.pl

**Keywords:** low-cycle fatigue, energy-based fatigue description, unified mechanics theory, anisotropy

## Abstract

**Highlights:**

**What are the main findings?**

- The research highlights the significant impact of S420M steel anisotropy resulting from rolling on the low-cycle fatigue life.
- The paper proposes and evaluates an energy accumulation graph as a generalization of isodamage lines for fatigue life prediction. It compares its effectiveness with the UMT thermodynamic approach.

**What are the implications of the main findings?**

- Fatigue life was consistently lower for samples taken perpendicular to the sheet surface compared to samples cut parallel to the rolling direction. This reduction in fatigue life varied significantly, from 40% to almost 290%, depending on the strain amplitude level.
- The thermodynamic UMT approach provides a physically grounded framework for fatigue damage prediction, integrating energy, entropy, and state variables into a single constitutive formulation.

**Abstract:**

This work investigates methods for predicting low-cycle fatigue life by employing new energy-based fatigue damage measures. The primary goal of this research is to evaluate whether fatigue life can be predicted based on an energy accumulation graph, proposed as a generalization of the isodamage lines concept. The efficiency of fatigue life predictions using this approach, derived from the empirical linear Palmgren–Miner hypothesis, is compared against the physically grounded Unified Mechanics Theory thermodynamic approach, which allows for general understanding of material degradation, in contrast to empirical approaches. The study also accounts for the influence of anisotropy resulting from the sheet rolling process on the fatigue response of S420M steel. Samples were tested in orientations both parallel to the rolling direction and perpendicular to the sheet surface. Microstructural analysis revealed a visible banded structure in the perpendicular samples, which is a consequence of anisotropy. The fatigue life of samples taken perpendicular to the sheet surface was lower than that of parallel samples. Verification of the linear Palmgren–Miner damage summation hypothesis, using both the classical fatigue chart and the cumulative energy chart, resulted in calculated fatigue life consistently higher than the experimental fatigue life in all cases. The reduction in fatigue life ranged from 40% (for total strain amplitude equal to 1.0%) to almost 290% for a strain amplitude of 0.25%. A comparative analysis of the unit loop energy shows that at all tested levels of strain amplitude, the unit loop energy of parallel samples is higher than that of samples perpendicular to the surface.

## 1. Introduction

Low-cycle fatigue (LCF) occurs under high strain amplitudes where significant plastic deformation takes place within a relatively low number of cycles. Predicting LCF life requires models that capture cyclic plasticity, energy dissipation, and material degradation (damage) mechanisms. From 1924 to the present day, approximately 40 different damage accumulation hypotheses have been developed [1,2,3]. The oldest and simplest hypothesis is the linear Palmgren–Miner (PM) hypothesis [4,5]. Various criteria can be used to assess fatigue damage accumulation, and multiple damage definitions may exist even within a single degradation mechanism. Researchers have characterized fatigue damage through several approaches: the cumulative cycle ratio [4,5], decreased Young’s modulus [6], diminished load-bearing capacity [7], crack propagation length [8], and accumulated strain energy [9]. Traditional strain-based methods, such as the Coffin–Manson relation, correlate fatigue life with plastic strain amplitude [10,11]. The energy approaches assume that cyclic plastic deformation produces irreversible energy dissipation, manifesting as heat and microstructural evolution, and fatigue failure is a process driven by progressive energy dissipation and damage accumulation. Multiple fatigue damage progression theories have been developed as alternatives to the PM model for energy-based characterization [12,13,14,15,16,17,18]. The first energy-based models, introduced by Landgraf [19] and Morrow [20], proposed correlating fatigue life directly with cyclic plastic strain energy. Later developments by Lemaitre and Chaboche [21] embedded energy dissipation into continuum damage mechanics (CDM), providing a theoretical basis for damage evolution laws dependent on dissipated energy. Hu et al. [22] refined these ideas by partitioning the total dissipated energy into initiation and propagation components, demonstrating strong correlations with experimental LCF data for structural steels. Tak et al. [23] extended the framework to include temperature and strain-rate effects for 316L stainless steel, illustrating improved life prediction accuracy under thermomechanical fatigue (TMF) conditions. Energy dissipated per cycle is quantified by the area within the stress–strain hysteresis loop. Analysis demonstrates that energy-based fatigue characterization offers greater comprehensiveness than stress- or strain-based methods because it captures stress–strain interaction effects.

Isodamage lines—representing constant damage—form the foundation of many fatigue damage hypotheses and are conventionally assumed to be linear. However, some studies report non-linear constant damage lines [1,18,24]. Hypotheses based on constant damage lines can be broadly categorized into two groups: those incorporating residual fatigue life considerations [1,25] and those founded on constant fatigue damage line existence [26,27]. Comparative analysis in [1] reveals that the linear PM hypothesis is interpretable using both constant fatigue damage lines and residual life line frameworks.

Degradation processes are irreversible, thermodynamically; all damage mechanisms are related to the dissipation of energy, because dissipation is a fundamental measure of irreversibility. Dissipation can be quantified by entropy generation. Thus, cumulative damage can be alternatively represented through dissipation or, equivalently, entropy generation [28,29,30]. For this reason, more recently, thermodynamically consistent frameworks such as the Unified Mechanics Theory (UMT) have emerged, integrating energy, entropy, and state variables into a single constitutive formulation [23,28,29,31,32,33,34]. The latter models establish a relationship between entropy production and damage defined as the relative change in disorder parameter, introduced by Boltzmann [35]. The Unified Mechanics Theory (UMT) builds upon the energy approach but anchors it in the laws of thermodynamics. In this context, fatigue damage corresponds to irreversible entropy generation and the evolution of a thermodynamic state variable, often expressed as the thermodynamic state index (TSI) [31]. Constitutive relations are derived from the first and second laws of thermodynamics, ensuring compatibility between mechanical dissipation, entropy production, and energy conservation.

Bin Jamal et al. [31] validated the UMT formulation for Ti–6Al–4V under cyclic loading, showing that entropy-based fatigue indicators captured cyclic softening, hardening, and life transitions. Similarly, Lee et al. [32,33,34] extended the UMT concepts to HCF regimes, confirming that entropy and TSI-based models could provide a continuous description across fatigue domains.

Both classical energy-based models and unified thermodynamic formulations view fatigue as an energy-driven degradation process, differing primarily in theoretical grounding and generality. The UMT offers a stronger physical foundation by linking macroscopic observables (stress, strain, energy dissipation) to microstructural thermodynamics. Conventional classical fatigue models incorporate material degradation from fatigue damage progression and energy dissipation through empirical dissipation and degradation evolution functions. These empirical functions necessitate curve-fitting to experimental data to determine parameters that often lack physical significance. Conversely, the UMT eliminates the requirement for empirically derived dissipation potentials obtained through curve-fitting to dissipation/degradation test data, as entropy generation is directly integrated into the structural differential equation. Nevertheless, the material’s thermodynamic fundamental equation must be analytically derived using fundamental physics and chemistry principles.

This work is a continuation of research on methods of low-cycle fatigue damage description [36,37,38,39]. Its primary goal is to answer the question of whether it is possible to predict fatigue life based on an energy accumulation graph as a generalization of the isodamage lines concept, and what the efficiency of fatigue life predictions for linear damage summation is in comparison with the UMT thermodynamic approach.

The experimental verification of the new methods carried out in this work may, in the future, allow for an improvement in the effectiveness of fatigue life calculations, and thus improve the safety of technical objects subjected to variable loads. This applies in particular to structural elements manufactured using unconventional methods such as additive manufacturing (AM) technologies. The manufacturing capabilities of additive methods are already very high, and the observed interest in this manufacturing technique practically guarantees that they will develop rapidly. This is evidenced, for example, by the growing number of research and implementation projects, as well as the number of publications on additive manufacturing techniques. This share will increase rapidly. A very important factor in favor of this is that the technological machines used in additive manufacturing are cheaper than cutting machine tools. New methods require non-standard testing methods, such as those based on an energy approach.

In addition, the work takes into account the influence of anisotropy resulting from the sheet rolling process on the fatigue response, on the example of S420M steel. The results of experimental tests of S420M steel for samples taken perpendicular to the sheet surface and parallel to the rolling direction are presented. The literature reports measurable differences in fatigue limits, fatigue life, and fatigue threshold behavior that stem from banding and pearlite scale. In such cases, energy metrics also have been used to quantify dissipation, though direct energy dissipation comparisons between banded and non-banded steels are reported less often [40]. The scope of the tests includes static tensile tests, low-cycle fatigue tests, and microstructure analysis. The test results are analyzed using both the classical and the UMT-based energy description of the fatigue process.

## 2. Materials and Methods

### 2.1. Isodamage Lines

**Figure 1:**
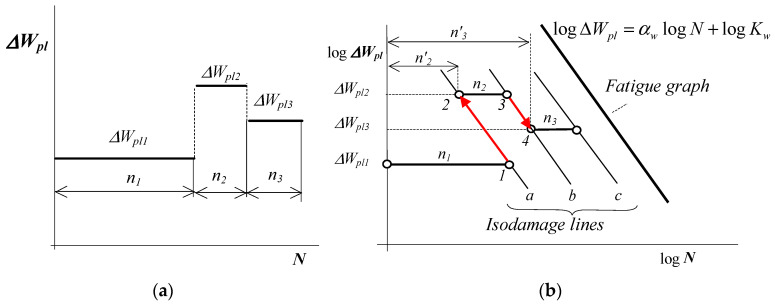
Isodamage lines in the PM hypothesis in terms of energy: (**a**) load program, (**b**) location of isodamage lines (in the double logarithmic scale).

The comparative study of damage cumulation techniques presented in [3] shows that residual fatigue life lines and permanent fatigue failure lines can serve as interpretive frameworks for the linear PM hypothesis. For explanatory purposes, Figure 1 schematically illustrates the damage accumulation process resulting from the isodamage line hypothesis connected to the PM hypothesis, quantified as plastic strain energy per cycle per unit volume of material, ${\Delta W}_{pl}$.

In this concept, the area below the fatigue curve (e.g., Wöhler’s curve) is divided by lines parallel to the fatigue curve (lines a, b, c in Figure 1). It is assumed that these lines define the locations where similar fatigue phenomena occur in the material during variable loading (e.g., for metals—plastic slips, slip bands, microcracks, etc.) at different load levels. For example, for a block load program (Figure 1a), after $n_{1}$ cycles (point 1 in Figure 1b) at the ${\Delta W}_{pl1}$ level, the degree of damage is the same as after ${n\prime }_{2}$ cycles at the ${\Delta W}_{pl2}$ level (point 2). After completing the next stage of the program, $n_{2}$ cycles (point 3), the degree of damage is the same as after completing ${n\prime }_{3}$ cycles at the constant level of ${\Delta W}_{pl3}$ (point 4). The last of these lines is the fatigue chart line indicating the occurrence of fatigue failure. The existence of these lines makes it possible to link the current load level with a specific number of cycles to the load history.

These lines are usually straight (e.g., Wöhler’s graph), although there are studies in which the isodamage lines are not straight [13,24]. Hypotheses based on the concept of isodamage lines can be divided into two groups. The first includes hypotheses that take residual strength into account [1,25]. The second are hypotheses based on the assumption of the existence of fatigue isodamage lines [26].

The diagram shown in Figure 1 was created under the assumption of constant plastic strain energy in a unit volume of material per cycle (unit energy ${\Delta W}_{pl}$) in each load cycle.

Despite employing new energy-based criterion variables, fatigue life calculation methods remain below anticipated efficiency levels for multiple reasons. One significant issue stems from the fact that plastic strain energy (${\Delta W}_{pl}$) is calculated post-test. Only a limited number of studies in the literature [9] maintain controlled plastic strain energy (${\Delta W}_{pl}=const$) throughout testing. Additionally, similar to other hysteresis loop parameters, the energy does not achieve complete stabilization under constant amplitude loading conditions, as confirmed by numerous investigations in the low-cycle metal fatigue regime [36,37,41,42]. Research is currently underway in controlled strain energy conditions [27], which offers an alternative to this approach.

The cumulative energy after n cycles, $\Sigma \Delta W_{pl}(n)$ is calculated as the summation of unit energies:

(1) $$\Sigma \Delta W_{pl}(n)=\sum_{j=1}^{n}\Delta W_{pl}(j)$$

where $\Delta W_{pl}(j)$ is the unit energy of the j-th cycle.

Recently, a number of papers have been published in which the authors propose a new fatigue characteristic called the energy accumulation curve [9,20,36,37,41,42]. Based on an extensive research program, the authors concluded that this could be the relationship between the cumulative energy $\Sigma \Delta W_{pl}$ in the sample until the moment of fracture as a function of the number of load cycles to failure. This relationship is described in a double logarithmic system by the following equation:

(2) $$log\Sigma \Delta W_{pl}(N)=\alpha_{\Delta W}\log N+logK_{\Delta W}$$

where N is the number of load cycles to failure, while $\alpha_{\Delta W}$ and $K_{\Delta W}$ are material parameters.

**Figure 2:**
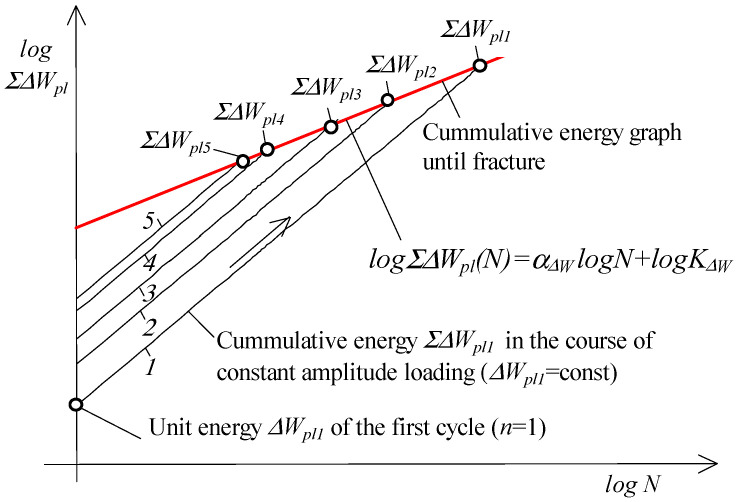
Creating an energy accumulation graph (1–5 denote the number of cycle).

The graph can be obtained on the basis of constant-amplitude fatigue tests (e.g., under conditions of $\varepsilon_{at}=const$ or $\sigma_{a}=const$) by summing the unit loop energy ${\Delta W}_{pl}$ in successive load cycles. According to the authors, the new characteristics can supplement the description of fatigue properties in terms of energy. Figure 2 shows a schematic representation of how to obtain the aforementioned characteristics.

**Figure 3:**
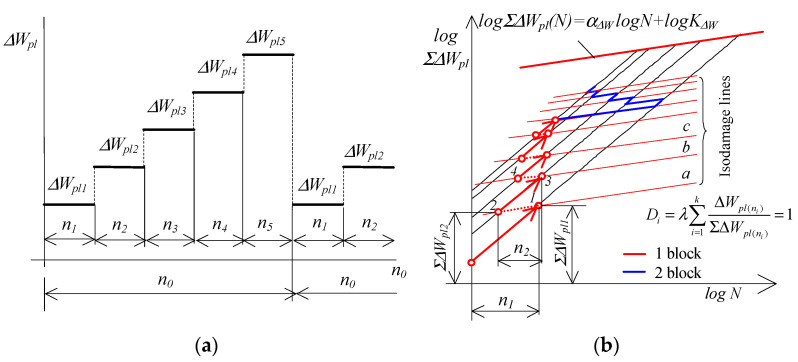
Summing up damage using a cumulative chart: (**a**) load program, (**b**) summing method.

It seems natural to ask whether the energy characteristics (Equation (2)) can be used as a basis for calculating fatigue life (summing up fatigue damage). In paper [13], the authors proposed a method of summing damage using the cumulative energy-based characteristic. In the proposed method, a certain difficulty is posed by determining the intersection point of the graph with the abscissa axis (Figure 2). The current paper attempts to transfer the assumptions formulated for Palmgren–Miner’s hypothesis to a cumulative graph. Figure 3 shows a schematic representation of the procedure for summing fatigue damage during the application of a gradually increasing load.

As in the classical concept of damage accumulation shown in Figure 1, the area under the accumulation curve described by Equation (2) is divided by lines parallel to the energy accumulation curve (lines a, b, c, Figure 3b). It is assumed that these lines indicate the same levels of material damage. Taking into account the results of cumulative energy studies published, among others, in [9,36,37,41,42], it is known that the lines introduced in this way cannot connect points with equal relative plastic strain energy increments. Larger damage increments occur at levels where the cumulative energy has a higher value. For example, for a block of a gradually increasing load program (Figure 3a), after $n_{1}$ cycles of the first stage of the program (point 1) at the level of ${\Delta W}_{pl1}$, the cumulative energy is ${\Sigma \Delta W}_{pl1}$. The cumulative energy in the second stage of the program (point 2) causing the same degree of damage is ${\Sigma \Delta W}_{pl2}$. The figure clearly shows that ${\Sigma \Delta W}_{pl2}<{\Sigma \Delta W}_{pl1}$. The further course of the procedure during the summation of damage is analogous to that in Figure 1. The last line of constant damage is the accumulation graph, indicating the occurrence of a macrocrack in the sample.

In the classical PM hypothesis (Figure 1), constant amplitude loading is assumed to produce equal damage contributions from each cycle. This implies that fatigue damage D after n constant amplitude loading cycles can be formulated as follows:

(3) $$D=\frac{n}{N}$$

In the case of a multi-stage load program (Figure 1), cracks are to be expected if the following condition is met

(4) $$D=\lambda \sum_{i=1}^{k}\frac{n_{i}}{N_{i}}=1$$

where λ is the number of repetitions of the load program block, *k* is the number of load levels in the program, $n_{i}$ is the number of cycles completed at the i-th level of constant amplitude load, and $N_{i}$ is the fatigue life at the i-th level of constant amplitude loading.

Using similar assumptions, but in relation to energy accumulation (using the accumulation graph, Figure 3), fatigue damage after n cycles of constant amplitude loading will be equal to the following:

(5) $$D=\frac{\Sigma \Delta W_{pl}\left(n\right)}{\Sigma \Delta W_{pl}(N)}$$

where $\Sigma \Delta W_{pl}\left(n\right)$ is the energy accumulated in the sample after n cycles of constant amplitude loading, and $\Sigma \Delta W_{pl}(N)$ is the energy accumulated in the sample until fracture.

Similarly to Equation (4), in the case of a multi-stage crack loading program, this should be expected if the following condition is met:

(6) $$D=\lambda \sum_{i=1}^{k}\frac{\Sigma \Delta W_{pl}(n_{i})}{\Sigma \Delta W_{pl}(N_{i})}=1$$

The cumulative dissipated energy up to sample failure in the fatigue test, $\Sigma \Delta W_{pl}(N)$, was computed as the summation of all hysteresis loop areas:

(7) $$\Sigma \Delta W_{pl}(N)=\sum_{j=1}^{N}\Delta W_{pl(j)}$$

For the total dissipated energy $\Sigma \Delta W_{pl}(N)$ calculations, the assumption is frequently made that identical unit energy corresponds to each hysteresis loop under constant amplitude loads. The total dissipated energy until material failure can therefore be established by multiplying the energy of one loop, $\Delta W_{pl(j)}$, from the material stabilization period (or from half the fatigue life j = 0.5N when stabilization is absent) by the cycles to failure, N:

(8) $$\Sigma \Delta W_{pl}(N)=N\cdot \Delta W_{pl}(j=0.5N)$$

**Figure 4:**
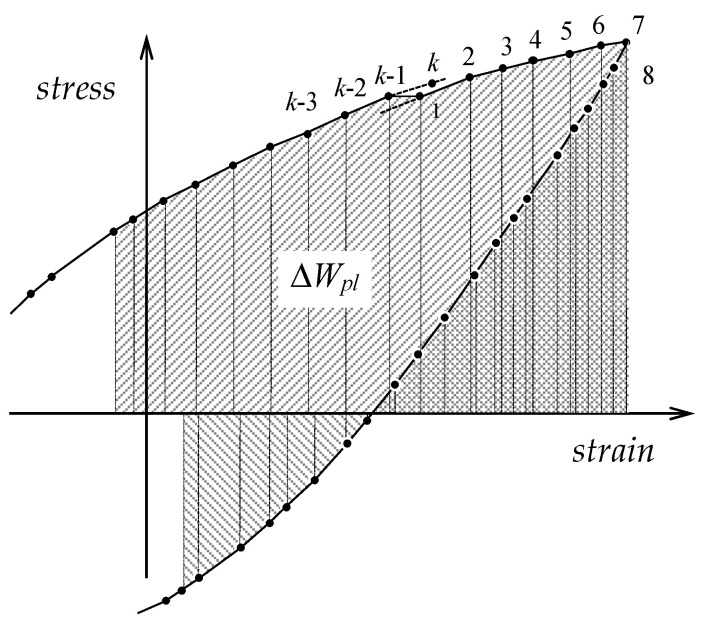
Scheme for numerical calculation of hysteresis loop area.

Stabilization of metals’ cyclic properties, however, is relatively rare, particularly under elevated temperature conditions. When materials do not exhibit cyclic stabilization (S420M steel being one such example), considerable differences emerge between energies calculated via Equations (7) and (8). The dissipated energy per cycle is quantified by the stress–strain hysteresis loop area (see Figure 4). For each individual j−th cycle, the energy $\Delta W_{pl}(j)$ was computed by numerical integration from the following formula:

(9) $$\Delta W_{pl}(j)=[\sum_{i=1}^{k-1}\frac{1}{2}(\sigma_{i}+\sigma_{i+1})(\varepsilon_{i+1}-\varepsilon_{i})]+(\sigma_{k}+\sigma_{1})(\varepsilon_{1}-\varepsilon_{k})$$

### 2.2. Generalization to UMT-Based Damage Description

The Unified Mechanics Theory provides a framework for understanding material degradation, particularly in the context of fatigue, by integrating classical mechanics with thermodynamics. It introduces a novel concept, the thermodynamic state index, to quantify degradation processes. The UMT unifies Newton’s universal laws of motion with the second law of thermodynamics at a fundamental level. This integration ensures that the governing differential equations for any system inherently include entropy generation, which is crucial for describing material degradation processes.

Per the second law of thermodynamics, damage progression must generate increased internal entropy production, which consequently functions as a damage indicator [29]. The total entropy $S_{RVE}$ of the representative volume element (RVE) quantifies disorder (thermodynamic probability) in the system using the relation derived by Boltzmann [35] ($k_{B}$ denotes the Boltzmann constant):

(10) $$S_{RVE}=k_{B}\ln W^{N_{RVE}}=N_{RVE}k_{B}\ln W$$

where W is the thermodynamic probability of one particle. The entropy per unit mass (specific entropy) is as follows:

(11) $$s=\frac{S_{RVE}}{m_{RVE}}=\frac{N_{RVE}k_{B}}{m_{RVE}}\ln W=\frac{R}{m_{s}}\ln W$$

where $m_{s}$ is molar mass and R is the gas constant.

The single particle thermodynamic probability is therefore as follows:

(12) $$W=e^{s\frac{m_{s}}{R}}$$

A measure of the thermodynamic state of particles contained in the volume of RVE may be defined as the relative change in disorder:

(13) $$\zeta =\frac{W-W_{0}}{W_{0}}=e^{∆s\frac{m_{s}}{R}}-1$$

where $\Delta s=s-s_{0}$ is the entropy generation. Variable ζ is related to the thermodynamic state index $\varphi =\frac{W-W_{0}}{W}$, defined by Basaran [28] in the following way:

(14) $$\zeta =\frac{\varphi }{1-\varphi }$$

The thermodynamic state variable ζ is a unitless quantity describing the difference in disorder between the original “ordered” state (or any reference state) and the current “disordered” state, related to the reference state, while φ relates the change in disorder to the current state.

As entropy generation may serve as a damage measure, a scalar damage variable, D, can be established in relation to the dissipative processes underlying entropy generation [30]:

(15) D=f(ζ)

Damage function (15) provides a transformation that maps entropy generation from the thermodynamics perspective to the damage perspective. In fatigue of ductile materials D, as the measure of degradation, can be defined, for example, as the drop in Young’s modulus, $D=1-\frac{E(D)}{E_{0}}$ [43], the reduction in load-bearing surface, $D=\frac{{dA}_{D}}{dA}$ [44], etc.

Based on the Boltzmann law, the averaged damage in the volume of RVE may be defined in the simplest form as proportional to the relative change in disorder [39]. Taking into account Equations (13) and (15), it can be written as follows:

(16) $$D=D_{0}\frac{W-W_{0}}{W_{0}}=D_{0}\left[e^{∆s\frac{m_{s}}{R}}-1\right]=D_{0}\zeta$$

where $D_{0}$ is a parameter. A fracture occurs when the damage level reaches the critical value $D_{cr}\leq 1$, analogically to (6). An equivalent indicator of fracture occurrence may be the critical value of the thermodynamic state variable, $\zeta_{cr}$, related to the critical value of entropy production in a material ${∆s}_{FFE}$. According to Equation (13), the fracture criterion may be expressed in the following form:

(17) $$\zeta =\zeta_{cr}=e^{\frac{{∆s}_{FFE}m_{s}}{R}}-1$$

where ${∆s}_{FFE}$ is the cumulative specific entropy at failure [45].

According to the UMT approach, the thermodynamic state variable φ (or, equivalently, ζ) serves as a linearly independent coordinate in addition to the classical space-time coordinate system (x,y,z,t). The thermodynamic lifespan of any closed system travels between zero and a critical value along the thermodynamic state axis, according to the second law of thermodynamics as formulated by Boltzmann.

In the case of plasticity as the only mechanical source of entropy production, the change in specific entropy, $\Delta s=s-s_{0}$, can be calculated from the following expression (ρ is the material density, θ is an absolute temperature, $\sigma_{ij}$ and $\varepsilon_{ij}^{p}$ respectively denote the components of Cauchy stress and plastic strain tensors, $k^{\theta }$ stands for thermal conductivity, while $r^{\theta }$ is the distributed internal heat source per unit mass):

(18) $$\Delta s=\int_{t_{0}}^{t}\frac{\sigma_{ij}\varepsilon_{ij}^{p}}{\theta \rho }dt+\int_{t_{0}}^{t}\frac{k^{\theta }{\left|grad\theta \right|}^{2}}{\theta^{2}\rho }dt+\int_{t_{0}}^{t}\frac{r^{\theta }}{\theta }dt$$

TSI is determined by entropy changes leading to predefined failure. Every dissipation process contributing to failure causes entropy to rise. Hence, a proper dissipation measure is needed for process life prediction. The present work considers solely mechanical dissipation processes under low-cycle fatigue loading conditions. Plastic dissipation is therefore treated as the dominant mechanism in these mechanical loading situations. Calculating entropy generation from plastic dissipation in mechanical loading experiments proceeds as follows:

(19) $$\Delta s=\int_{t_{0}}^{t}\frac{\sigma_{ij}\varepsilon_{ij}^{p}}{\theta \rho }dt$$

Since the entropy is an additive quantity, the UMT-based degradation description is also based on the calculation and summation of the hysteresis loops’ area. However, a linear damage summation hypothesis is adopted in the classical PM approach, Equation (4), or in the extended cumulative energy approach expressed by Equation (6), which does not apply in this case. The contributions to the thermodynamic state index, resulting from the subsequent loading cycles, do not linearly add. This is a qualitative difference between the classical linear summation hypotheses and the UMT approach. It should also be underlined once again that the exponential function in the TSI definition is not an assumption/hypothesis, but it follows from the Boltzmann law. The UMT is therefore physically grounded and allows for a general understanding of the material degradation, in contrast to empirical approaches. In our paper, we investigate and compare these energy-based and thermodynamic approaches, highlighting the benefits of the UMT, while also exploring the utility of energy accumulation graphs derived from the Palmgren–Miner hypothesis.

### 2.3. Material and Experimental Testing

#### 2.3.1. Material Characterization

**Figure 5:**
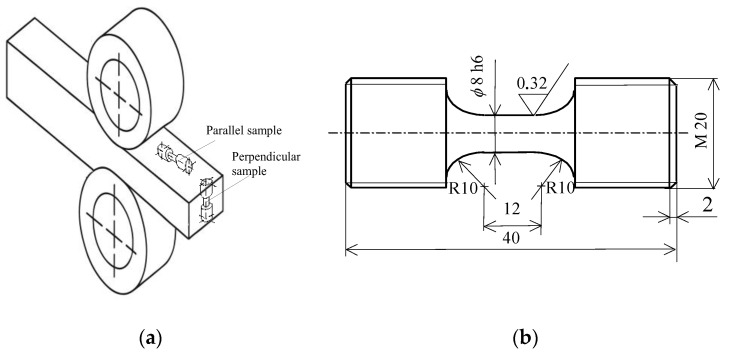
(**a**) Anisotropy directions in relation to the rolling direction, (**b**) sample dimensions.

Based on an analysis of literature data [38], it can be concluded that mechanical properties are most often determined for material samples taken from sheets parallel to the rolling direction. As a result, the through-thickness direction, i.e., the direction perpendicular to the sheet surface, is usually neglected, despite its critical importance for assessing material anisotropy and susceptibility to lamellar cracking. This is explained in Figure 5. The samples cut out in the rolling direction will be called “parallel samples”, while those cut perpendicular to the sheet surface will be called “perpendicular samples”.

The anisotropy of the mechanical properties of metal alloys (especially rolled ones) perpendicular to the surface causes, in some design situations, the risk of so-called lamellar cracks [46]. This applies in particular to welded or threaded joints.

In order to evaluate the fatigue properties of samples oriented perpendicular to the sheet surface (Figure 5a), test samples were taken from a 40 mm thick steel sheet with the following chemical composition (wt %) determined using a Bruker Q4 Tasman spark spectrometer (Bruker Corporation, Karlsruhe, Germany): C (0.16); Mn (1.53); Si (0.22); S (0.009); P (0.018).

The material used for manufacturing the samples was in the form of cuboids measuring 20 mm × 20 mm × 40 mm, cut from a 40 mm-thick sheet of S420M steel. To avoid the influence of processing on the mechanical properties, the sheet was cut with a water jet. After cutting the cuboids, the measuring and gripping parts of the samples were shaped during metal turning in accordance with Figure 5. After the turning operation, the measuring part of the samples was subjected to grinding. The roughness obtained was $R_{a}=0.32$. The appropriate dimensions of the samples for static and fatigue testing were prepared in accordance with the guidelines of the standard [46] (Figure 5b).

#### 2.3.2. Static Tests

An Instron 8502 testing machine was employed for static testing. Due to the 40 mm sheet metal thickness, the total sample length for static and fatigue tests corresponded to this thickness. Samples manufactured per standard [46] fully satisfied the dimensional specifications recommended for low-cycle testing [38]. Figure 5 presents the sample locations on the sheet and test sample dimensions.

Real-time load force and sample elongation data were recorded during static tests. Sample elongation was monitored using a static extensometer (type 2630-110, IMT, High Wycombe—Buckinghamshire, UK) with a 10 mm gauge length, +100% measuring range, and attachment to the sample’s gauge section. A force transducer (2518-113, IMT, High Wycombe—Buckinghamshire, UK) with a ±100 kN range measured the applied force. All tests occurred at 21 °C. Sample separation within the gauge section served as the static test termination criterion. Three replicate static tensile tests were performed for each sample orientation.

#### 2.3.3. Low-Cycle Fatigue Tests

**Figure 6:**
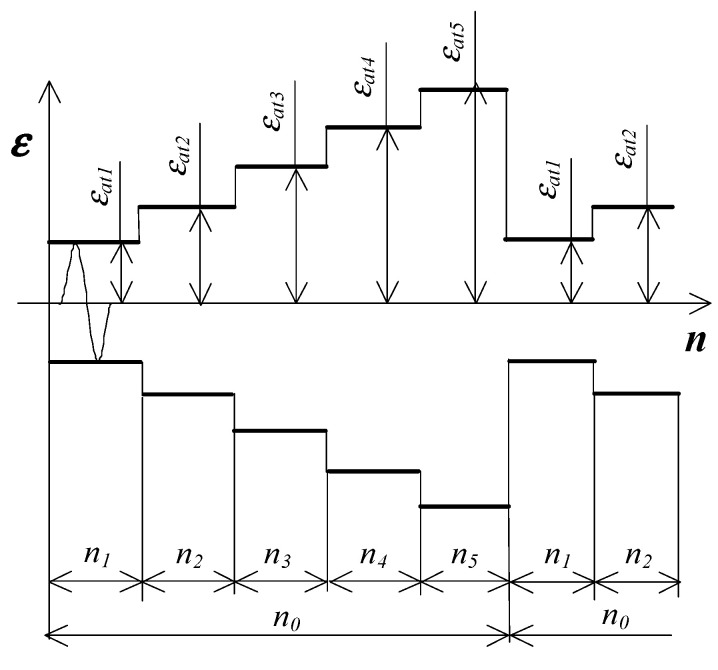
Variable loads scheme.

Low-cycle tests consisted of subjecting samples to a constant amplitude load and a programmed load with a frequency of 0.2 Hz. Figure 6 shows the variable load programs.

Low-cycle tests were carried out with controlled deformation conditions ($\varepsilon_{at}=const$). Strain amplitude levels $\varepsilon_{at}$ for both loading programs were established after evaluating static tensile test data. The following strain amplitude levels were used in tests: $\varepsilon_{at1}=0.25\%$; $\varepsilon_{at2}=0.35\%$; $\varepsilon_{at3}=0.5\%$; $\varepsilon_{at4}=0.8\%$; $\varepsilon_{at5}=1.0\%$ (Figure 6). In constant-amplitude tests, real-time force and specimen deformation measurements were captured for designated load cycles. Force was quantified using a load cell (2518-113) having a ±250 kN range. Specimen deformation was assessed via a dynamic extensometer (type 2630-110) possessing a 10 mm gauge length, ±10% measurement range, and attachment to the specimen’s gauge area. Following standard [46], fatigue test completion was defined by hysteresis loop deformation (kink) in the compression half-cycle. Three replicate constant-amplitude fatigue tests occurred at each deformation level. In programmed loading scenarios (Figure 6), each of the five stages involved 10 load cycles. Upon completing $n_{0}=50$ cycles, load blocks repeated until complete specimen separation in the measurement region. Full load program blocks were recorded throughout testing. Three fatigue tests per sample orientation were executed under programmed loading. Both constant-amplitude and programmed tests proceeded at 20 °C using an Instron 8502 hydraulic testing machine (IMT, High Wycombe—Buckinghamshire, UK).

## 3. Results and Discussion

### 3.1. Static Tensile Tests

The results of static tensile tests are discussed in detail in [38] and will not be repeated here. Based on the analysis of these results, a reduction in plastic properties, i.e., Z reduction and A elongation, was found in samples oriented perpendicular to the sheet surface. A comparative analysis of the results allows us to conclude that the Z reductions are significantly larger than those required by the standard, and thus the steel meets the requirements specified in standard [46] for the risk of lamellar cracks.

### 3.2. Fatigue Testing

#### 3.2.1. Constant Amplitude Loads

**Figure 7:**
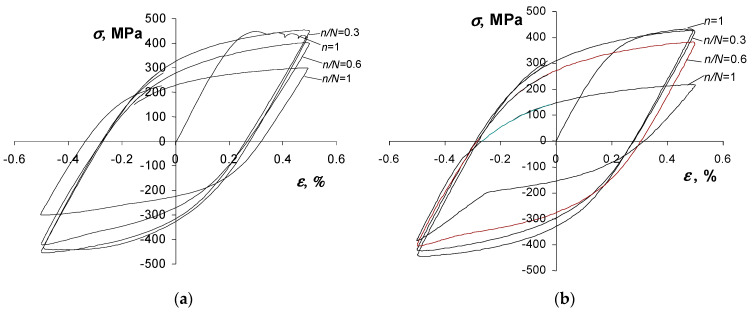
Chosen hysteresis loops for $\varepsilon_{at}$ = 0.5%: (**a**) parallel samples, (**b**) perpendicular samples.

The results of low-cycle constant-amplitude tests were compiled in accordance with the procedures specified in standard [46]. In this study, two hysteresis loop parameters were used to analyze the test results, i.e., stress $\sigma_{a}$ and plastic strain $\varepsilon_{ap}$. The results of the tests in terms of strain are presented, among others, in [38]. The following study focuses on the description of low-cycle properties in terms of energy. The instantaneous values of the force applied to the sample and its deformation recorded during the tests allowed the value of plastic deformation energy to be determined for each load cycle. The instantaneous stress in the sample, σ, was calculated as the ratio of the instantaneous force applied to the sample to the initial cross-sectional area of the sample. Due to the criterion value adopted in this study (dissipated energy ${\Delta W}_{pl}$), Figure 7 shows, for example, the recorded hysteresis loops of (parallel to the rolling direction) samples at a single deformation level $\varepsilon_{at}=0.5\%$.

**Figure 8:**
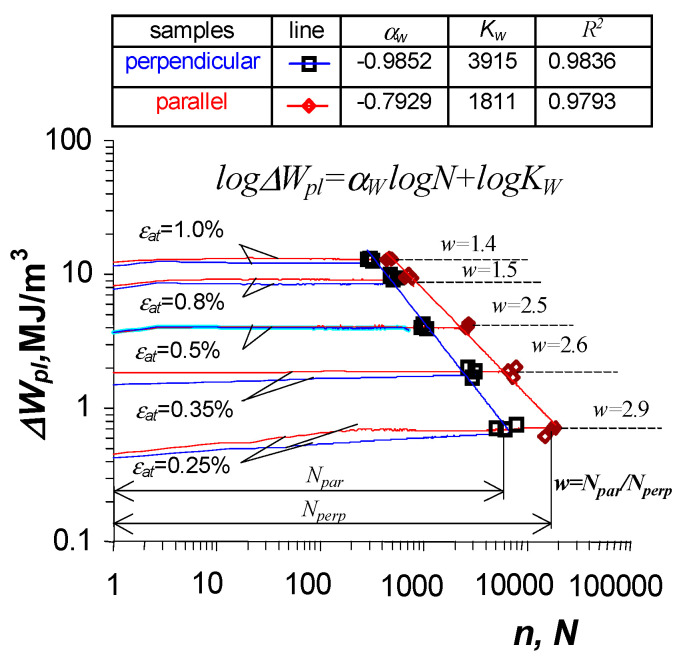
Fatigue diagrams in terms of energy.

Based on the analysis of the position of successive hysteresis loops, it can be concluded that S420M steel undergoes slight softening during testing. This can be concluded both on the basis of the position of successive hysteresis loops (Figure 7) and on the basis of an analysis of changes in plastic deformation energy ${\Delta W}_{pl}$ as a function of the number of load cycles (Figure 8). The figure also shows points illustrating the fatigue life N obtained at given deformation levels. The fatigue life results were approximated by regression equations of the following form:

(20) $$log\left(\Delta W_{pl}\right)=\alpha_{w}\log N+{logK}_{w}$$

Based on the analysis of the results shown in Figure 8, it can be concluded that the orientation of the sample has a clear impact on fatigue life. A comparative analysis of average fatigue lives shows that the fatigue life of perpendicular samples is lower than that of parallel samples. The magnitude of the reduction in fatigue life is not constant and depends on the level of deformation. Figure 8 shows the values of the quotients illustrating the ratio of fatigue life of parallel samples, $N_{par}$ in relation to the fatigue life of perpendicular samples, $N_{perp}$ ($w=N_{par}/N_{perp}$). Based on these results, it can be concluded that the reduction in fatigue life varies from 40% (for a level of $\varepsilon_{at}=1.0\%$) to almost 290% (for a level of $\varepsilon_{at}=0.25\%$). A comparative analysis of changes in unit loop energy $\Delta W_{pl}$ allows concluding that at all levels of strain amplitude, the energy of the parallel samples is higher than the energy of the samples perpendicular to the surface. The study analyzed the accumulation of plastic strain energy $\Delta W_{pl}$ at five strain amplitude levels (Figure 6).

**Figure 9:**
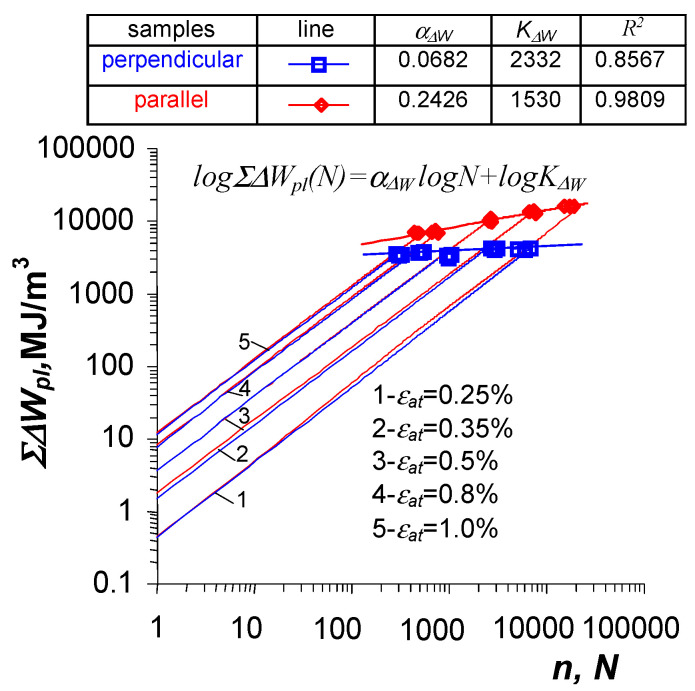
Energy accumulation curves under constant amplitude loads.

Figure 9 shows the values of accumulated energy $\Sigma \Delta W_{pl}$ in samples with different orientations until fracture. The values of accumulated energy $\Sigma \Delta W_{pl}$ are described by a regression equation of the form (2).

Despite the small variation in unit plastic strain energy $\Delta W_{pl}$ in a single load cycle (see Figure 8), the plastic strain energy $\Sigma \Delta W_{pl}$, accumulated until fracture in the perpendicular samples is significantly lower than the energy accumulated in the parallel samples.

**Figure 10:**
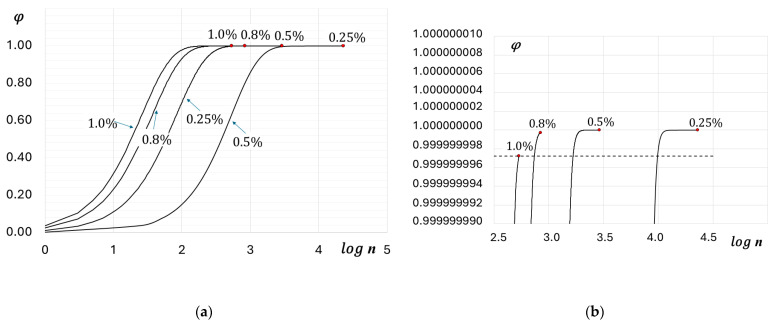
(**a**) Evolution of thermodynamic state index φ versus the decimal logarithm of the number of cycles, (**b**) enlarged section of the graph showing critical TSI values. Numbers near curves indicate strain amplitudes.

As far as the fatigue degradation is considered in terms of the internal energy dissipation, the fatigue life prediction can alternatively be performed with the use of a more general UMT approach. Figure 10 shows the evolution of the thermodynamic state index φ proposed by Basaran [28], for parallel samples. The TSI, by definition, asymptotically approaches unity (Figure 10a). As can be observed in Figure 10b, experimentally determined critical values of the TSI, $\varphi_{cr}$, reach values very close to 1, which is consistent with the understanding of the critical fatigue damage value, Equation (6). In several papers [32,33] it is proposed that the critical value $\varphi_{cr}$ is user-defined, and it needs to be set according to the problem at hand. However, the accuracy of determining $\varphi_{cr}$ has a significant impact on the predicted low-cycle fatigue life. For example, if a constant value of $\varphi_{cr}=0.9999999972$ is set for all strain amplitudes $\varepsilon_{at}$ (see Figure 6), then the error in predicted fatigue life will be 12% for $\varepsilon_{at4}=0.8\%$, 42% for $\varepsilon_{at3}=0.5\%$, and even as much as 57% for $\varepsilon_{at1}=0.25\%$.

**Figure 11:**
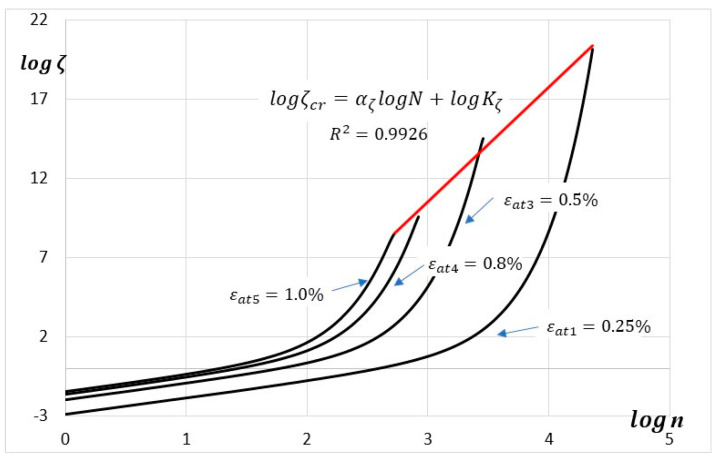
Evolution of thermodynamic state variable ζ versus the number of cycles (double logarithmic scale) for parallel samples.

On the other hand, the thermodynamic state variable ζ, defined by Equation (13) can take any positive value and is not bounded above (see Figure 11). As in the case of the cumulative energy (see Figure 9), in a double logarithmic coordinate system, the critical values $\zeta_{cr}$ fulfill the equation of a straight line:

(21) $$log\zeta_{cr}=\alpha_{\zeta }logN+logK_{\zeta }$$

with coefficients $\alpha_{\zeta }=7.2477$ and $K_{\zeta }=10^{-11.2040}$ (the coefficient of determination $R^{2}=0.9926)$.

**Figure 12:**
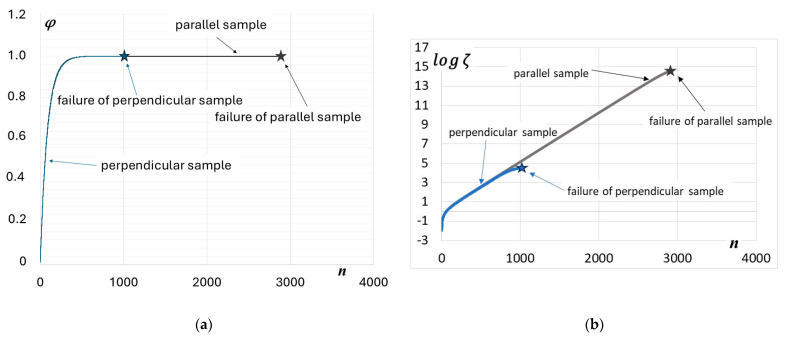
Comparison of thermodynamic state variable evolutions for parallel and perpendicular samples ($\varepsilon_{at3}=0.5\%$): (**a**) variable φ, (**b**) variable ζ.

When comparing the service life of parallel and perpendicular samples, an additional benefit of using the thermodynamic state variable ζ becomes apparent, as it can clearly demonstrate the differences in fatigue life resulting from the sheet rolling process. Figure 12 shows the comparison of thermodynamic state evolution curves for parallel and perpendicular samples resulting from tests under strain amplitude $\varepsilon_{at}=0.05$. As can be seen in Figure 12a, the index φ does not exhibit practically any difference between the two samples, neither in the course of φ evolution, nor in the critical value $\varphi_{cr}$. However, in the case of ζ (Figure 12b) a significant difference, especially in the critical value $\zeta_{cr}$, is detected.

#### 3.2.2. Programmed Loads

**Figure 13:**
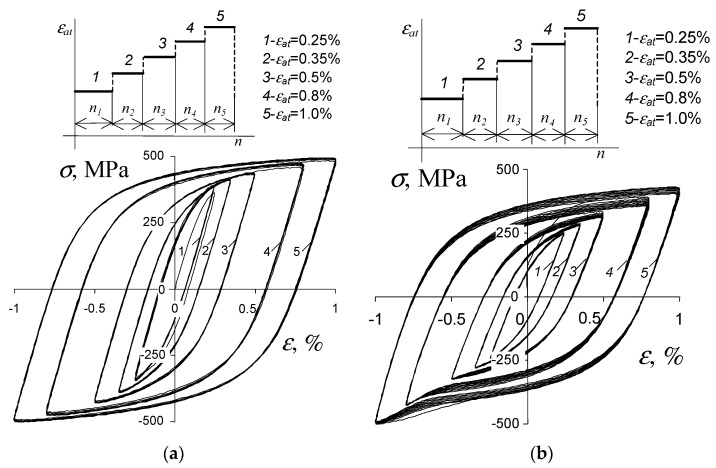
Hysteresis loops in a single load block: (**a**) first block, (**b**) last block before sample fracture.

Similarly to constant amplitude loads, under programmed load conditions, a very slight influence of sample orientation on cyclic properties defined by hysteresis loop parameters was observed. For this reason, the graphical presentation of the test results was limited to parallel samples only. Figure 13 shows the hysteresis loop graphs in the first and last repetitions of the load program block, for a parallel sample.

**Figure 14:**
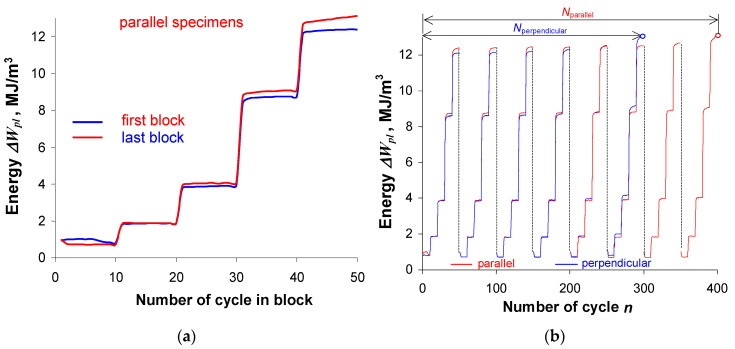
Unit loop energy $\Delta W_{pl}$: (**a**) in the program block, (**b**) as a function of the number of cycles.

Similarly to the constant amplitude tests, during the analysis of programmed load results, a comparison was made of changes in unit energies $\Delta W_{pl}$ dissipated in a parallel and perpendicular sample (Figure 5). The slight changes and variations in energy at individual deformation levels observed during constant amplitude loading (Figure 8) were also visible during programmed loading. In order to compare the changes in energy $\Delta W_{pl}$ during programmed loading, Figure 14a shows the changes in energy in one load block at different sample life stages: in the first block—halfway through the fatigue life period, and in the last block—just before sample fracture. Figure 14b shows exemplary graphs of energy $\Delta W_{pl}$ as a function of the number of load cycles in subsequent blocks of the load program.

**Figure 15:**
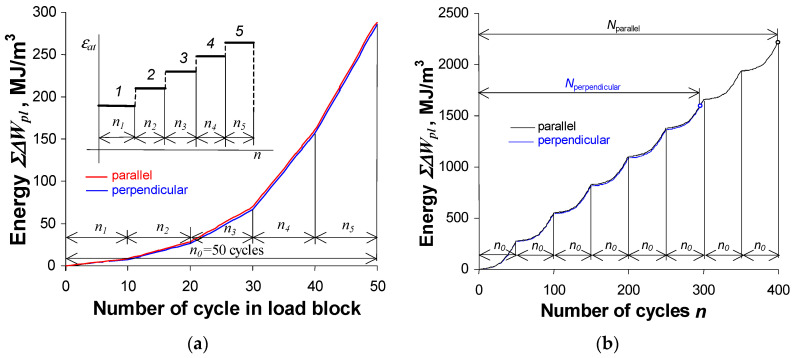
Cumulative energy $\Sigma \Delta W_{pl}$: (**a**) in one load block (numbers 1–5 denote load levels), (**b**) as a function of the number of cycles.

A comparative analysis of the energy $\Delta W_{pl}$ at the same strain amplitude levels shows that, similarly to the constant amplitude loading (Figure 8), the energy $\Delta W_{pl}$ in samples perpendicular to the rolling direction is only slightly lower than the energy $\Delta W_{pl}$ obtained for parallel samples. A slightly greater variation in energy is more evident at the highest deformation levels ($\varepsilon_{at}=0.8\%$ and 1.0%). Similarly to constant amplitude loading, the energy accumulation during programmed loading was also analyzed. Figure 15 shows the energy accumulation graphs in the first block of the load program and in the entire test.

The slight differences in unit energy obtained for perpendicular and parallel samples result in very similar energy accumulation curves. This is also confirmed by the analysis of the energy accumulated in subsequent blocks of the programmed load (Figure 15b). Despite the similar unit energy values, the energy accumulated until fracture in samples perpendicular to the rolling direction is significantly lower than the energy accumulated in parallel samples. This is the result of a banded ferrite–pearlite microstructure resulting from rolling, and altering how cyclic work is converted to dissipated energy by concentrating strain in the softer ferrite bands and at phase interfaces.

### 3.3. Banded Microstructure Effects

The accumulated plastic strain energy $\Sigma \Delta W_{pl}$ is adopted in this study as a macroscopic measure of irreversible mechanical dissipation associated with cyclic plastic deformation. In the low-cycle fatigue regime of ductile steels, the area of the stress–strain hysteresis loop $\Delta W_{pl}$ directly represents the plastic work dissipated per loading cycle, which has been confirmed in recent energy-based fatigue studies using cumulative plastic strain energy as a life-controlling parameter [9,22]. Microstructural anisotropy does not alter the physical meaning of $\Delta W_{pl}$, but affects the spatial distribution of plastic deformation and local dissipation mechanisms. In banded ferrite–pearlite microstructures, cyclic deformation becomes localized within the softer ferrite bands and at ferrite–pearlite interfaces, leading to enhanced local energy dissipation and earlier microcrack initiation. As a result, samples with different banding orientations reach failure at different critical values of $\Sigma \Delta W_{pl}$, indicating that this quantity is a microstructure- and orientation-dependent damage indicator rather than a universal material constant.

**Figure 16:**
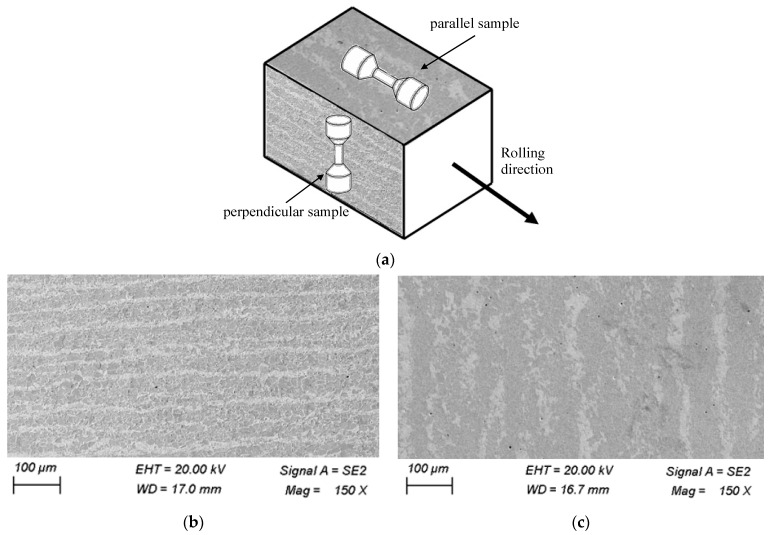
Microstructure of the sample material: (**a**) position of the sample in the sheet, (**b**) parallel sample microstructure, (**c**) perpendicular sample microstructure.

In the present research, the microstructure of samples in a cross-section parallel to the sheet surface (perpendicular samples) and perpendicular to the sheet surface (parallel samples) was analyzed. The cross-section orientations are explained in Figure 16a, while images of the microstructure are shown in Figure 16b,c.

The tested steel is characterized by a ferritic-pearlitic structure with a dominant ferrite content. An unfavorable, banded arrangement of pearlite oriented in the rolling direction was observed. The width of the pearlite bands ranges from approx. 5 µm to approx. 25 µm, depending on the direction of sampling—perpendicular to the sheet surface and parallel to the rolling direction. The occurrence of an unfavorable banded structure is most often related to the chemical composition heterogeneity of the shaped element [47,48]. A clear relationship between the orientation of the sample and the results of fatigue tests has been observed.

#### 3.3.1. Microstructural Mechanisms Affecting Fatigue Behavior

The microstructural mechanisms that convert cyclic loading into dissipated energy include dislocation activity in ferrite, cementite fracture/voiding in pearlite, and incompatible strains at ferrite–pearlite interfaces; these together determine crack nucleation and growth behavior.

Dislocation accumulation in ferrite produces intragranular slip bands, extrusion/intrusion features, and substructure evolution that enlarge hysteresis loops and raise $\Delta W_{pl}$ locally [49,50]. Pearlite fracture and void formation (cementite lamellae failure and void coalescence) generate internal damage that consumes plastic energy and can produce internal cracks that accelerate failure at high strain amplitudes [51]. Interface incompatibility at ferrite–pearlite boundaries concentrates stresses and is a preferred nucleation site for small cracks, so the interface both dissipates energy during repeated opening/closing and serves as a low-toughness path for crack advance [40,52]. In papers [53,54], the authors observed that cracks are mainly initiated at the ferrite–pearlite interface. The heterogeneity of the chemical composition in the banded structure adversely affects the material’s ability to prevent crack propagation, which has a negative impact on the fatigue strength of the component. In particular, the adverse effect of banded structure on fracture resistance is observed at low temperatures [55]. The direction of crack propagation is of significant importance from the point of view of the fatigue strength of ferritic-pearlitic steels. Cracks propagating parallel to the bands have a negative effect on fatigue strength. On the other hand, cracks oriented perpendicular to the bands can increase fatigue strength due to the ability of hard pearlite to block crack propagation [56].

#### 3.3.2. Banding Orientation and Spacing

The banded structure clearly affects the anisotropy of mechanical properties. Banding orientation and the spacing/scale of pearlitic lamellae strongly influence how much cyclic energy the microstructure can absorb before macro-crack growth. Parallel loading tends to allow cracks to propagate along ferrite first and then along ferrite–pearlite interfaces, enabling long, straight growth with lower branching and lower crack-retarding dissipation [57]. On the other hand, perpendicular loading produces intense branching and crack deflection at band boundaries, increasing tortuosity and thereby raising energy consumed per unit crack growth [57].

Fine pearlite/small interlamellar spacing promotes cyclic softening and more uniform dislocation distribution, which can reduce local ${\Delta W}_{pl}$ per cycle, but raise high-cycle fatigue limits [49]. In contrast, coarse pearlite/larger spacing favors cyclic hardening, formation of dislocation cells, and greater localized plastic work in ferrite, resulting in different energy absorption patterns and often shorter HCF/LCF lives depending on strain amplitude [49,58]. The effect of spacing/orientation is load-amplitude dependent: the same band geometry may increase life at one strain amplitude (by deflecting cracks) and decrease it at another (by promoting localization) [49,58].

#### 3.3.3. Quantitative Interpretation of Band Width Effects

The observed pearlite band widths in the range of approximately 5 µm to 25 µm provide a quantitative microstructural scale that governs strain localization and fatigue damage evolution. Experimental and numerical studies have shown that decreasing band spacing increases strain incompatibility between ferrite and pearlite, leading to higher local plastic strain amplitudes and elevated dissipation rates at ferrite–pearlite interfaces under cyclic loading conditions [53,54]. Zhang et al. [53] demonstrated that banded microstructures promote strong localization of plastic strain within ferrite bands, with crack nucleation preferentially occurring at ferrite–pearlite interfaces once a critical local strain energy density is exceeded. Similarly, Narasaiah and Ray [54] reported that band widths of the order of several micrometers act as effective strain concentrators, accelerating small crack initiation in low-carbon steels.

From an energetic perspective, narrower band spacing enhances local plastic work per unit volume, resulting in higher local $\Delta W_{pl}$ values even when nominal hysteresis loop parameters remain comparable. This explains why samples with different banding orientations reach fracture at different critical values of accumulated plastic strain energy $\Sigma \Delta W_{pl}$. Studies on banded steels have further shown that increased strain localization associated with finer band spacing leads to reduced fatigue life and earlier crack initiation under the low-cycle fatigue conditions [49,57]. Therefore, the lower fatigue durability observed for perpendicular samples in the present study can be quantitatively interpreted as a consequence of enhanced strain localization and accelerated energy dissipation driven by the characteristic band width scale of the ferrite–pearlite microstructure.

### 3.4. Experimental Verification of P-M Linear Damage Summation Hypothesis

The fatigue life of perpendicular samples under programmed loads was lower than that of parallel samples, both in constant-amplitude tests and programmed load tests. To verify Palmgren–Miner’s linear damage summation hypothesis, the fatigue life graphs in the ${\Delta W}_{pl}-N$ coordinate system (described by Equation (20) and shown in Figure 8), as well as energy accumulation graphs in the ${\Sigma \Delta W}_{pl}-N$ system (described by Equation (2) and shown in Figure 9), were used. The procedure for summation was explained in Figure 1 and Figure 3, and the calculation results are summarized in Table 1.

**Figure 17:**
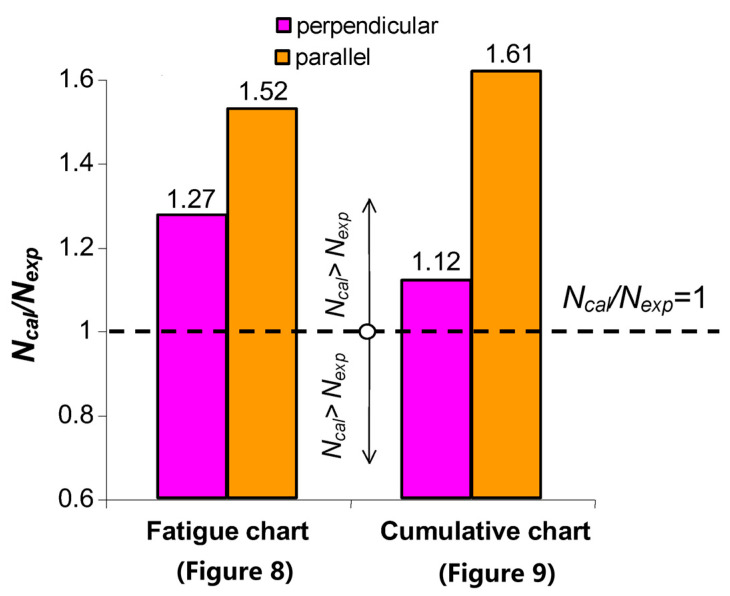
Fatigue life predictions obtained from PM linear damage summation and the energy accumulation-based method.

Based on a comparative analysis of the calculation results, it can be concluded that regardless of the type of characteristics used, the fatigue life results obtained from the calculations are similar. In both cases, the fatigue life obtained from the calculations $N_{calc}$ is higher than the fatigue life $N_{exp}$ obtained from experimental tests. The above results are also illustrated in Figure 17.

## 4. Conclusions

Examining plastic deformation energy ${\Delta W}_{pl}$ changes relative to the number of load cycles demonstrates that S420M steel shows minor cyclic property variations for both sample orientations. In the present paper, the energy-based descriptions were used to confirm the significant impact of sample orientation on fatigue life. Despite comparable hysteresis loop parameters, perpendicular samples are characterized by lower fatigue life compared to parallel samples. The reduction in fatigue life applies to both constant-amplitude and programmed loads. The results obtained confirm the findings described, among others, in [38].

The development of fatigue testing methods, particularly testing equipment, allows for real-time measurement of hysteresis loop parameters and analysis of fatigue progression using the criterion value of plastic deformation energy ${\Delta W}_{pl}$. The research results also confirm the possibility of describing fatigue properties using the characteristics of the cumulative energy ${\Sigma \Delta W}_{pl}-N$ coordinate system.

Fatigue life calculations performed using the cumulative energy characteristics naturally complement the current methods of fatigue life calculation. The results of fatigue life calculations obtained using the classic fatigue curve (Figure 8) and the accumulation curve (Figure 9) exhibit small differences.

As the material degradation is a manifestation of entropy production, the energy-based description of fatigue life is here generalized to the entropy production-based UMT approach. The thermodynamic state variable describing the material degradation process is here compared with the thermodynamic state index proposed by Basaran [28]. This approach, combining Newtonian mechanics and thermodynamics, has a qualitative advantage over classical approaches. In Newtonian continuum mechanics, degradation evolution is modeled using empirical functions obtained by curve-fitting a function to degradation test data. Using such an approach, the thermodynamic consistency is lost, and the models become case-specific (for example, the degradation evolution function/potential obtained from a low-frequency loading does not apply to high-frequency loading). In addition, empirical evolution functions commonly require extensive curve-fitting parameters devoid of physical significance when dissipative mechanisms multiply. Unified Mechanics Theory combines Newton’s universal laws of motion and the second law of thermodynamics at the ab initio level. Since entropy generation is intrinsically included in material equations, empirical functions derived by curve-fitting dissipation/degradation test data are unnecessary. However, analytical derivation of the material’s entropy production equation based on fundamental physics and chemistry principles is mandatory. For low-cycle fatigue conditions, plasticity is the predominant entropy production source.

From an industrial application perspective, the proposed energy-based methodology can be directly applied to fatigue life assessment of structural components manufactured from rolled steel plates, particularly when loading occurs in the through-thickness direction. The procedure requires performing a limited number of low-cycle fatigue tests on specimens extracted along representative material orientations and determining the $\Delta W_{pl}-N$ and $\Sigma \Delta W_{pl}-N$ characteristics. The critical accumulated plastic strain energy $\Sigma \Delta W_{pl}$ can then be adopted as a failure criterion. In practical engineering applications, measured or numerically simulated service load histories may be converted into incremental plastic strain energy contributions and cumulatively summed until the critical energy threshold is reached, enabling fatigue life estimation without the need for long-term full-scale testing. This approach is particularly relevant for welded structures and joints susceptible to lamellar cracking, where conventional stress- or strain-based criteria do not adequately capture the effects of microstructural anisotropy.

## Figures and Tables

**Table 1:** Fatigue life results from calculations and tests.

No.	Sample Type	$\mathrm{Average}\;\mathrm{Fatigue}\;\mathrm{Life}\;\mathrm{from}\;\mathrm{Tests}\;N_{exp}$	$N_{exp}$ (Mean)	$\mathrm{Fatigue}\;\mathrm{Life}\;\mathrm{from}\;\mathrm{Calculations}\;N_{calc}$			
				Basis for Calculations			
				Fatigue Chart (Figure 8)	$N_{calc}$ $/N_{exp}$	Cumulative Chart (Figure 9)	$N_{calc}$ $/N_{exp}$
1	perpendicular	305	290	371	1.27	327	1.12
2		288					
3		278					
4	parallel	445	413	632	1.52	669	1.61
5		404					
8		391					

## Data Availability

The original contributions presented in this study are included in the article. Further inquiries can be directed to the corresponding author.

## Abbreviations

The following abbreviations are used in this manuscript:

PM	Palmgren–Miner
LCF	Low-cycle fatigue
HCF	High-cycle fatigue
CDM	Continuum damage mechanics
TMF	Thermomechanical fatigue
TSI	Thermodynamic state index
UMT	Unified Mechanics Theory
RVE	Representative volume element
